# Aspin: neurosurgical aspirin intervention prognostic study — perioperative continuation versus discontinuation of aspirin in lumbar spinal surgery, a randomized controlled, noninferiority trial

**DOI:** 10.1186/s13063-024-07945-w

**Published:** 2024-02-29

**Authors:** Ahmed Zian, Gijsbert M. Overdevest, Pieter J. Schutte, Frederikus A. Klok, Ewout W. Steyerberg, Wouter A. Moojen, Niels A. van der Gaag

**Affiliations:** 1 Department of Neurosurgery, Haaglanden Medical Center, The Hague, The Netherlands; 2 Department of Neurosurgery, Leiden University Medical center (LUMC), Leiden, The Netherlands; 3 Department of Neurosurgery, Haga Teaching Hospital, The Hague, The Netherlands; 4 Department of Vascular Internal Medicine, Leiden University Medical Center (LUMC), Leiden, The Netherlands; 5 Department of Clinical Biostatistics and Medical Decision Making, Leiden University Medical Center (LUMC), Leiden, The Netherlands

## Abstract

**Rationale:**

Aspirin is typically discontinued in cranial and spinal surgery because of the increased risk of hemorrhagic complications, but comes together with the risk of resulting in an increase of cardiac and neurologic thrombotic perioperative events.

**Objective:**

The aim of this study is to investigate the non-inferiority of perioperative continuation of aspirin patients undergoing low complex lumbar spinal surgery, compared with the current policy of perioperative discontinuation of aspirin.

**Study design:**

A randomized controlled trial with two parallel groups of 277 cases (554 in total).

**Study population:**

Patients undergoing low complex lumbar spinal surgery and using aspirin. All patients are aged >18 years*.*

**Intervention:**

Peri-operative continuation of aspirin.

**Study outcomes:**

Primary study outcome: composite of the following bleeding complications:

- Neurological deterioration as a result of hemorrhage in the surgical area with cauda and/or nerve root compression.
- Post-surgical anemia with hemoglobin level lower than 5 mmol/l, requiring transfusion.
- Subcutaneous hematoma leading to wound leakage and pain higher than NRS=7.
- Major and/or minor hemorrhage in any other body system according to the definition of the International Society on Thrombosis and Haemostasis bleeding scale.

Secondary study outcomes:

- Each of the individual components of the primary outcome
- Absolute mean difference in operative blood loss between the study arms
- Thrombo-embolic-related complications:
- Myocardial infarction
- Venous thromboembolism
- Stroke
- Arterial thromboembolism

**Further study outcomes:**

Anticoagulant treatment satisfaction by the Anti-Clot Treatment Scale (ACTS) and general health by the Patient-Reported Outcomes Measurement Information System (PROMIS Global-10) in the pre- and postoperative phase.

**Nature and extent of the burden and risks associated with participation, benefit, and group relatedness:**

Participation in this study imposes no additional risk to patients. Currently, there is no consensus on whether or not aspirin should be discontinued before cranial or spinal surgery. Currently, aspirin is typically discontinued in cranial and spinal surgery, because of a potential increased risk of hemorrhagic complication. An argument not based on a clinical trial. However, this policy might delay surgical procedures or carry the risk of resulting in an increase in cardiac and neurologic thrombotic perioperative events. It is unclear if the possibility of an increase in hemorrhage-related complications outweighs the risk of an increase in cardiac and neurologic thrombotic perioperative events.

Furthermore, the Data Safety Monitoring Board (DSMB) will be asked for safety analysis by monitoring the study.

There are no further disadvantages to participating in this study. Outcome measurements are recorded during admission and regular outpatient visits, and thus, do not require additional visits to the hospital.

## Introduction and rationale

Current guidelines regarding the safety of the perioperative (dis)continuation of aspirin for surgical procedures fail to provide clear recommendations regarding patients undergoing cranial and spinal surgery. Currently, aspirin is discontinued in cranial and spinal surgery because of a potential increased risk of hemorrhagic complications. However, this policy might delay surgical procedures and carries the risk of resulting in an increase in cardiac and neurologic thrombotic perioperative events. It is unclear if the possibility of an increase in hemorrhage-related complications outweighs the risk of an increase in cardiac and neurologic thrombotic perioperative events. Therefore, a randomized controlled trial is required.

Cardiovascular diseases (CVDs) are the primary cause of death in developed countries and are expected to be the leading cause of death worldwide [1]. Cardiovascular diseases are a group of illnesses including myocardial infarction, coronary heart disease, and stroke. In the primary and secondary prevention of CVDs anticoagulant drugs play a key role, with aspirin being the most commonly used [1–3]. Specifically for the Netherlands, aspirin is prescribed for circa 230,000 patients annually [4]. With the increasing incidence of CVDs, the usage of anticoagulant drugs will increase forming a problem during surgical intervention.

Currently, there is no consensus on whether aspirin should be discontinued before neurosurgical intervention. One of the arguments leading the discussion is the POISE-2 trial by Devereaux et al., in which the conclusion was that procedural continuation of aspirin did not reduce the rates of all-cause mortality of non-fatal myocardial infarction. But it does come together with increased risks of major bleedings [5].

These results are supported by two reviews that included mostly non-RCT studies.

A review and meta-analysis by Burger et al. in non-cardiac surgery showed a significant increase of procedural bleeding complications, without a significant reduction nor increase of death or nonfatal myocardial infarctions. A low dose of aspirin did not significantly differ in bleeding risk or complications or mortality related to it [6]. The same was concluded in the review of Kiberd and Hall [7].

Another interesting pharmacological phenomenon is the aspirin withdrawal rebound effect, the hyperactivity of platelets after the interruption of aspirin [8]. Even though there is limited knowledge supporting this effect, it leads to clinical caution to discontinue aspirin. Multiple cases in different surgical fields describe fatal thrombo-embolic complications after discontinuing aspirin perioperatively. Limited knowledge is available about the pharmacokinetic availability of aspirin after interruption and the regaining of platelet function thereafter. A small study by Alcock et al. showed, in healthy participants, no evidence for a rebound phenomenon which leaves this argument open for discussion [9].

These results are contrasted by the study of Oscarsson et al. [10]. This randomized, double-blinded, placebo-controlled trial studied the effects of discontinuing aspirin on the occurrence of major adverse cardiac events together with hemorrhagic complications in a diversity of surgical interventions. This study showed a significant reduction in cardiac events perioperatively, a risk reduction of 7.2%, and no difference in hemorrhagic complications were found. Note bene, this difference is statistically not significant because of an early termination of inclusion. Additionally, this difference is based on mainly patients undergoing abdominal, urologic, orthopedic, and gynecologic surgery that are known as immobile patients post-operatively leading to a higher thrombo-embolic risk and are not completely comparable to neurosurgical patients undergoing spinal surgery which is often improving mobility by pain reduction and motor control. In addition to these results, the CLASP study, a gynecological randomized trial studying the effects of low-dose aspirin in the prevention and treatment of pre-eclampsia in pregnant women, did not observe an increase in uterine, placental or fetal hemorrhagic complications in the study group [11]. Unfortunately, these studies did not include any neurosurgical cases. This restricts the little evidence for continuing aspirin in neurosurgical cases and leads to more interest and concern in this matter.

A systematic review and meta-analysis regarding the safety of aspirin continuation in spinal surgery was conducted by our research group [12]. Only three non-randomized studies, including 370 patients undergoing cervical, thoracic, and lumbar spine surgery were identified. No significant differences in mean perioperative blood loss were seen between the aspirin-continuing group and the aspirin-discontinuing group. Similar non-significant differences between the two groups were found for cardiac events, stroke, and surgical site infections [13–15].

In addition to spinal surgery, the evidence in cranial surgery is even more limited. A comparative study by Rahman et al. in 83 patients undergoing craniotomy for brain tumor demonstrated no increased risk of perioperative hemorrhage-related complications among patients continuing aspirin [16]. Additionally, an observational study by Palmer at all, found no association between aspirin and postoperative hemorrhage [17]. In case of cerebral aneurysmatic pathology, aspirin is recommended for unruptured aneurysms and prevents ruptures by its anti-inflammatory effects [18]. In a ruptured aneurysm with subarachnoidal hematomas continuation versus discontinuation of aspirin did not lead to significant differences in bleeding risk, bleeding-related complications, or prevention of secondary ischemia due to vasospasms [19, 20].

In conclusion, there is a paucity of studies regarding the safety of the continuation of aspirin during cranial and spinal procedures, and the available evidence is of low methodologic quality. The hypothesis is that potential bleeding complications in cranial and spinal surgery might exceed the risks reported in other surgical literature. Especially in cranial surgery, postoperative bleeding may be catastrophic due to the confined space and vulnerability of the surrounding structures, but no studies exist to support this hypothesis. The current clinical practice in patients on aspirin and due to undergo a cranial or spinal is to discontinue aspirin 5 days prior to surgery. There is no clinical evidence to support this policy. Therefore, we propose a randomized controlled, non-inferiority study comparing the perioperative (dis)continuation of aspirin in spinal surgery.

## Objectives

### Primary study outcome: composite of the following bleeding complications

- Neurological deterioration as a result of hemorrhage in the surgical area with cauda and/or nerve root compression.
- Post-surgical anemia with hemoglobin level lower than 5 mmol/l, requiring transfusion.
- Subcutaneous hematoma leading to wound leakage and pain higher than NRS=7.
- Major and/or minor hemorrhage in any other body system according to the definition of the International Society on Thrombosis and Haemostasis bleeding scale.

### Secondary study outcomes

- Each of the individual components of the primary outcome
- Absolute mean difference in operative blood loss between the study arms
- Thrombo-embolic-related complications:
- Myocardial infarction
- Venous thromboembolism
- Stroke
- Arterial thromboembolism

### Primary outcomes: haemorrhage-related complications

#### Neurological deterioration as a result of hemorrhage in the surgical area with cauda and/or nerve root compression

The incidence of 30-day postoperative reoperation incidence is recorded in the hospital information system and recorded with a case record form at discharge from the hospital and during postoperative outpatient clinic visits. Of particular interest are directly hemorrhage-related reoperations. The aforementioned surgical site hemorrhages and postoperative subcutaneous, epidural or subdural hematomas are infrequent complications of spinal surgery. In case these hemorrhages excerpt pressure on the surrounding neurologic structures (e.g., cauda equine, nerve roots, or peripheral nerves) a rapid evacuation of the hematoma is required. Furthermore, perioperative hemorrhage-related complications can also result in an indirect need for reoperation. For instance, suboptimal nervous tissue decompression or tumor removal due to excessive bleeding during surgery might result in a need for reoperation. Therefore, the overall 30-day reoperation rate is recorded and compared among both treatment groups.

#### Post-surgical anemia with hemoglobin level lower than 5 mmol/l, needing transfusion

In case of peri- and/or post-operative blood loss leading to symptomatic anemia, objectified with laboratory research, with a hemoglobin level lower than 5 mmol/l a hemoglobin transfusion will be indicated.

Excessive perioperative blood loss can require postoperative allogeneic blood transfusions. The postoperative need for allogenic blood transfusions is registered in the hospital information system and is recorded with a case record form at discharge from the hospital.

The use of autologous blood transfusion is restricted to patients undergoing extensive surgical procedures with high expected blood loss. At the discretion of the surgeon or the anesthesiologist, a cell-saver autologous blood recovery system can be used during surgery. Blood recovered by this system is considered perioperative blood loss. In case a significant amount of blood is recovered and the patient is likely to benefit from an autologous transfusion, autologous blood will be transfused back to the patient.

#### Subcutaneous hematoma leading to wound leakage and pain score

Hemorrhage-related complications include surgical site hemorrhages and postoperative subcutaneous, epidural, or subdural hematomas. Hemorrhage-related complications resulting in an increased length of hospital stay or that require either invasive or non-invasive treatment are recorded in the hospital information system and are recorded with a case record form that is to be filled out at the time of discharge from the hospital and during postoperative outpatient clinic visits. Pain scores will be registered as well, as subcutaneous hematomas can lead to increased discomfort. Pain scores higher than 7 according to the Numeric Rating Scale (NRS) will be registered in the database. Potentially, some complications of spinal surgery can also be indirectly attributed to hemorrhage-related complications. For instance, surgical site hematomas are associated with an increased incidence of postoperative infections. Therefore, the overall 30-day complication rate is recorded and compared among both treatment groups.

#### Hemorrhage in any other body system

Hemorrhage in any other body system within 30 days post-surgery will be noted and compared between groups. These will be classified according to the International Society on Thrombosis and Haemostasis bleeding scale (ISTH-scale) [21].

### Secondary objective

#### All separate primary outcomes individually

##### Absolute mean difference in operative blood loss between the study arms

Perioperative blood loss is determined by measuring blood recovered in the suction device during surgery and weighting of blood-saturated gauzes used during surgery. The Validated Intraoperative Bleeding Scale (VIBe scale) will be used in order to objectify the intraoperative bleeding severity [22]. All cases will receive a vacuum drainage system applied to the surgical wounds in order to record the postoperative blood loss in the first 24 h. Blood loss is registered in the hospital information system and recorded with a case record form that is to be filled out at the time of discharge from the hospital.

##### Thrombo-embolic-related complications

*Myocardial infarction:* myocardial ischemic events diagnosed by a cardiologist according to the fourth universal definition of myocardial infarction.

*Stroke:* diagnosed by a neurologist defined as an acute or transient neurological deterioration with a positive radiological finding for a cerebral ischemic event.

*Venous thromboembolism:* diagnosed by a vascular internist defined as a concordant clinical presentation with a modified Wells score higher than 3, a positive D-dimer level > ng/mL, and a positive ultrasonographical examination.

##### Arterial thromboembolism

All 30-day complications perioperative after spinal surgery are recorded. The incidence of all thrombo-embolic events is of interest in order to assess to compare the occurrence between the control and study group. During hospital stay and at regular postoperative outpatient clinical appointments within 6–12 weeks after surgery all perioperative complications are recorded and evaluated in a standardized manner using a case record form. Complications are classified according to a hospital database thesaurus and the severity of the complication is graded (see Table 1).

**Table 1:** Grading of complications according to Clavien-Dindo

Grade	Description
1A	Recovery after non-invasive treatment (e.g., medication, physical therapy)
1B	Recovery after invasive treatment (except for operation in an OR) or admission to intensive care
2	Recovery after (re-)operation in an OR
3A	Complication still persists or is treated at the time of registration
3B	Complication resulting in permanent loss of function or disability
4	Death

### Study design

The study is a randomized controlled, non-inferiority trial with two parallel groups. Patients are randomly allocated to either perioperative continuation of aspirin or discontinuation group prior to spinal surgery. Perioperative blood loss, hemorrhage-related complications, need for reoperation events and cardiovascular and/or neuro-embolic events, within 30 days after surgery are evaluated in both treatment groups.

All patients of 18 years and older, referred to the outpatient clinic of the Department of Neurosurgery who are scheduled for elective spinal surgery are eligible for inclusion. The hospitals participating in this study are as follows:

- Haaglanden Medical Center
- HAGA Teaching Hospital Den Haag
- Spaarne Gasthuis Haarlem
- Alrijne Hospital Leiderdorp

## Study population

### Population and feasibility

All patients undergoing low complex lumbar spinal surgery in an elective setting using aspirin as antithrombotic therapy.

In the evaluation of the importance of aspirin as an antithrombotic drug and its relevance in its current and future usage, Dutch national statistics show a stable number of 228,000 to 230,000 prescriptions between 2013 and 2017 [4]. In the current cardiologic guidelines in the Netherlands, aspirin forms an important drug in the so-called double platelet therapy in combination with clopidogrel. Studying perioperative continuation and discontinuation of aspirin is relevant and is expected to stay in use.

### Inclusion criteria

In order to be eligible to participate in this study, a subject must meet all of the following criteria:

- Scheduled low complex lumbar spinal surgery defined as removal of intervertebral disc herniation, decompression of lumbar canal, and/or foraminal stenosis.
- Preoperative use of aspirin
- Age >18

### Exclusion criteria

A potential subject who meets any of the following criteria will be excluded from participation in this study:

- Spinal oncology
- Staged surgeries lasting more than one day
- Patients with a pre-existing coagulopathy (a proven hemophilia and/or thrombocyte function disorder)
- Patients using antithrombotic drugs or other platelet aggregation inhibitors than aspirin
- Patients with absolute contraindications for discontinuing aspirin (e.g., coronary stenting within 1 year)
- Patients aged under 18
- Emergency surgical procedures
- Incompetence to decide, i.e., in case of severe cognitive impairment or psychiatric illness.

### Sample size calculation

#### Sample size calculation

Because of the research question, the noninferiority of the continuation of aspirin perioperatively in comparison to discontinuation, a noninferiority setup is chosen. Since hemorrhage risk is the main risk of interest in the intervention groups, the sample size calculation is based on these risk percentages. The thrombo-embolic complications are not expected to be raised in the intervention group, as the aspirin functions as a prevention for these events to occur.

The sample size for a noninferiority approach was estimated as *n*=554, *n*=277 per study arm. This was based on the following hemorrhage risk percentages: 1% for the control group, 3% for the intervention group, and 6.1 percentage points as the non-inferiority margin. Calculations were made in PASS Sample Size Software, setting a non-inferiority trial with binary parameters. Below is a summary statement for the sample size estimation.

#### Summary statement

The current practice in Dutch hospitals is to discontinue aspirin pre-operatively. Annual complication registration in our regional database shows a 1% bleeding risk after spinal surgery with the current management. The study by Rahman et al. had a postoperative hematoma rate of 8% versus 4%, with a difference of 4% when not on aspirin perioperatively while Soleman et al. had a 2.5% in postoperative hemorrhagic complications when continuing aspirin [15, 16]. The POISE2-trial presents a hemorrhage risk of 4.6% in patients continuing aspirin in overall surgical patients [5]. These numbers were assumed to not be representative as the studies and the outcome parameters were not comparable to the Aspin study and the study population was not equivalent. Our spinal expert opinion in the Haaglanden Medical Center recommends to maintain a percentage of 3% as a more realistic bleeding risk for the study group.

Sample sizes of 277 in group one and 277 in group two achieve 80% power to detect a

Non-inferiority margin difference between the group proportions of 0.0610. The reference group proportion is 0,0100. The treatment group proportion is assumed to be 0.0710 under the null hypothesis of inferiority. The power was computed for the case when the actual treatment group proportion is 0,.0300. The test statistic used is the one-sided Score test (Miettinen and Nurminen). The significance level of the test was targeted at 0.0250.

## Treatment of subjects

### Investigational product/treatment

#### Standard of care

The group that discontinues aspirin is considered receiving usual care. Currently, all scheduled spinal procedures are performed after discontinuing aspirin for 5 days. After discontinuation of aspirin, coagulation is presumed to have returned to normal because of thrombocyte renewal every 7–10 days. Routinely, no laboratory measurements of blood coagulation are performed to assure this assumption.

Patients who require emergency surgery and thus rapid correction of coagulopathy are excluded from participation.

Aspirin is regularly resumed 3 days after surgery. Exceptions are made at the discretion of the surgeon.

In case of a thrombotic cardiac or neurologic event, perioperative treatment is provided as a conventional matter. Generally, a cardiologist or neurologist is consulted and in close agreement with the surgeon antithrombotic therapy is resumed, and/or other antithrombotic agents are administered.

#### Investigational treatment

The intervention group consists of patients who continue aspirin perioperative. Patient continue their regular dosage of aspirin. The most common indications for the prescription of aspirin are as follows:

- Primary and secondary prophylaxis of myocardial infarction
- Primary and secondary prophylaxis of stroke (Transient Ischemic Attack and Cerebrovascular Accident)
- Prevention of vascular graft occlusion
- Prevention of (hemodialysis) shunt occlusion

The regular dosage for both cardiac and neurologic indications is 80 or 100 mg daily. Patients using antithrombotic drugs or other platelet aggregation inhibitors than aspirin are excluded from the study.

### Use of co-intervention

In this study, all other medications will be continued according to the standard of care. In case the patient uses other anti-thrombotic medication than aspirin, inclusion must not be considered.

In case the study will be extended with the inclusion of patients undergoing spinal lumbar fusion surgery, the following is of interest:

Currently, patients undergoing a spinal spondylodesis with or without posterior lumbar interbody fusion, receive pre-operative 1 g of tranexamic acid on a standard base. This will remain unchanged, considering no bias will occur as patients will be blindly randomized in the control or study group.

### Escape medication

In case aspirin is continued, a potential need for co-interventions exists if hemorrhage-related complications with an indication for reoperation occur. Depending on the severity of the complication and the assessment of the treating physician, thrombocyte transfusion or medication may be administered for rapid correction of coagulopathy.

In case aspirin is discontinued, no co-intervention is to be expected. Patients who require emergency surgery and thus rapid correction of coagulopathy through the administration of platelets or medication are excluded from participation.

Both groups of patients receive venous thromboembolism prophylaxis by means of subcutaneous injections with low molecular weight heparin as standard of care. As this applies to both treatment groups and is not related to the prevention of cardiac and neurologic thrombotic perioperative events, this is not to be considered a relevant co-intervention.

The same management will be maintained in case the study is extended with patients undergoing lumbar spinal fusion. Then the management will be maintained for the administration of tranexamic acid peri-operatively, which is administered per protocol in the posterior lumbar interbody fusion procedures (PLIF). Assuming that patients will be randomly assigned in both study groups, this will remain unchanged.

## Investigational product

Not applicable.

## Non-investigational product

Not applicable.

## Methods

### Primary study outcome: composite of the following bleeding complications

- Neurological deterioration as a result of hemorrhage in the surgical area with cauda and/or nerve root compression.
- Post-surgical anemia with hemoglobin level lower than 5 mmol/l, requiring transfusion.
- Subcutaneous hematoma leading to wound leakage and pain higher than NRS=7.
- Major and/or minor hemorrhage in any other body system according to the definition of the International Society on Thrombosis and Haemostasis bleeding scale.
- Postoperative hemorrhage leading to a clinical indication for re-operation (c.q. evacuation of hematoma).

### Secondary study outcomes

- Each of the individual components of the primary outcome
- Absolute mean difference in operative blood loss between the study arms
- Thrombo-embolic-related complications:
- Myocardial infarction
- Venous thromboembolism
- Stroke
- Arterial thromboembolism

### Primary outcomes: hemorrhage-related complications

#### Neurological deterioration as a result of hemorrhage in the surgical area with cauda and/or nerve root compression

The incidence of 30-day postoperative reoperation incidence is recorded in the hospital information system and recorded with a case record form at discharge from the hospital and during postoperative outpatient clinic visits. Of particular interest are directly hemorrhage-related reoperations. The aforementioned surgical site hemorrhages and postoperative subcutaneous, epidural, or subdural hematomas are infrequent complications of spinal surgery. In case these hemorrhages exert pressure on the surrounding neurologic structures (e.g., spinal cord, cauda equine, nerve roots, or peripheral nerves) a rapid evacuation of the hematoma is required. Furthermore, perioperative hemorrhage-related complications can also result in an indirect need for reoperation. For instance, suboptimal nervous tissue decompression or tumor removal due to excessive bleeding during surgery might result in a need for reoperation. Therefore, the overall 30-day reoperation rate is recorded and compared among both treatment groups.

#### Post-surgical anemia with hemoglobin level lower than 5 mmol/l, needing transfusion

In case of peri- and/or post-operative blood loss leading to symptomatic anemia, objectified with laboratory research, with a hemoglobin level lower than 5 mmol/l a hemoglobin transfusion will be indicated.

Excessive perioperative blood loss can require postoperative allogeneic blood transfusions. The postoperative need for allogenic blood transfusions is registered in the hospital information system and is recorded with a case record form at discharge from the hospital.

The use of autologous blood transfusion is restricted to patients undergoing extensive surgical procedures with high expected blood loss. At the discretion of the surgeon or the anesthesiologist, a cell-saver autologous blood recovery system can be used during surgery. Blood recovered by this system is considered perioperative blood loss. In case a significant amount of blood is recovered and the patient is likely to benefit from an autologous transfusion, autologous blood will be transfused back to the patient.

#### Subcutaneous hematoma leading to wound leakage and pain score

Hemorrhage-related complications include surgical site hemorrhages and postoperative subcutaneous, epidural, or subdural hematomas. Hemorrhage-related complications resulting in an increased length of hospital stay or that require either invasive or non-invasive treatment are recorded in the hospital information system and are recorded with a case record form that is to be filled out at the time of discharge from the hospital and during postoperative outpatient clinic visits. Pain scores will be registered as well, as subcutaneous hematomas can lead to increased discomfort. Pain scores higher than 7 according to the Numeric Rating Scale (NRS) will be registered in the database. Potentially, some complications of spinal surgery can also be indirectly attributed to hemorrhage-related complications. For instance, surgical site hematomas are associated with an increased incidence of postoperative infections. Therefore, the overall 30-day complication rate is recorded and compared among both treatment groups.

#### Hemorrhage in any other body system

Hemorrhage in any other body system within 30 days post-surgery will be noted and compared between groups. These will be classified according to the International Society on Thrombosis and Haemostasis bleeding scale (ISTH-scale) [21].

### Secondary objective

#### All separate primary outcomes individually

##### Absolute mean difference in operative blood loss between the study arms

Perioperative blood loss is determined by measuring blood recovered in the suction device during surgery and weighting of blood-saturated gauzes used during surgery. The Validated Intraoperative Bleeding Scale (VIBe scale) will be used in order to objectify the intraoperative bleeding severity [22]. All cases will receive a vacuum drainage system applied to the surgical wounds in order to record the postoperative blood loss in the first 24 h. Blood loss is registered in the hospital information system and recorded with a case record form that is to be filled out at the time of discharge from the hospital.

##### Thrombo-embolic-related complications

*Myocardial infarction:* myocardial ischemic events diagnosed by a cardiologist according to the fourth universal definition of myocardial infarction.

*Stroke:* diagnosed by a neurologist defined as an acute or transient neurological deterioration with a positive radiological finding for a cerebral ischemic event.

*Venous thromboembolism:* diagnosed by a vascular internist defined as a concordant clinical presentation with a modified Wells score higher than 3, a positive D-dimer level > ng/mL, and a positive ultrasonographical examination.

##### Arterial thromboembolism

All 30-day complications perioperatively after spinal surgery are recorded. The incidence of all thrombo-embolic events is of interest in order to assess to compare the occurrence between the control and study groups. During hospital stay and at regular postoperative outpatient clinical appointment within 6–12 weeks after surgery, all perioperative complications are recorded and evaluated in a standardized manner using a case record form. Complications are classified according to a hospital database thesaurus and the severity of the complication is graded (see Table 1).

### Other study parameters

In order to assess potential confounders additional patient data will be assembled. Body weight, smoking behavior, comorbidities and indication for aspirin prescription, pre-operative use of non-steroid anti-inflammatory drugs (NSAIDs) as analgesic.

Furthermore, the pre- and postoperative satisfaction with the anticoagulant treatment will be assessed by the Anti-Clot Treatment Scale (ACTS). The general health will be assessed pre- and postoperatively by the Patient-Reported Outcomes Measurement Information System (PROMIS Global-10 version 1.2 Dutch) [23, 24].

### Randomization, blinding, and treatment allocation

After evaluation of inclusion and exclusion criteria by the neurosurgeon participants will be randomized by block randomization via Castor EDC. Neither patients of surgeons are blinded to treatment allocation. For practical considerations, patients cannot be blinded to treatment allocation, as they are required to continue or discontinue their aspirin. Surgeons and other treating physicians are not blinded to treatment allocation because the management of potential complications requires knowledge of all current medications. Furthermore, the robust outcome measurements of the study are unlikely affected by knowledge of treatment allocation.

### Study procedures

Patients eligible for inclusion are enrolled at the outpatient clinic. Patients who are scheduled for low-complex lumbar spinal surgery, meeting inclusion and exclusion criteria are informed about the study by their physician. Patients will receive written information on the purpose and the conduction of the study and a consent form. Patients are enabled to carefully read the information on the study after the initial visit. They are allowed to withdraw from the study at any time. Patients who did not give their permission during the initial visit or want to reconsider their decision can contact the research nurse.

Randomization is performed when the operation is scheduled. Regularly, this takes place after pre-surgery evaluation of the patient by the anesthesiologist and time on the potential waiting list prior to surgery. As the standard of care is to discontinue aspirin (and other antithrombotic drugs or other platelet aggregation inhibitors than aspirin) this is at least 1 week prior to surgery.

Study data is recorded with case record forms. After receiving consent from the patient demographic and clinical characteristics are recorded. Outcome measurements are recorded at discharge from the hospital and during the postoperative visit to the outpatient clinic. During this visit, all 30-day postoperative complications will be recorded, that not have been recorded at discharge previously.

No additional visits to the outpatient clinic are required for the purpose of the study.

### Withdrawal of individual subjects

#### Specific criteria for withdrawal

Subjects can leave the study at any time for any reason if they wish to do so without any consequences. The investigator can decide to withdraw a subject from the study for urgent medical reasons.

Patients are removed from the study in case the operation is canceled after a patient has been randomized. Patients who receive new antithrombotic prescriptions in the waiting time to the operation will be withdrawn. Logically, patients who do not follow up on study instructions as consent will be excluded.

### Replacement of individual subjects after withdrawal

Inclusion will continue until 554 patients in toto are reached, and patients withdrawing consent will be replaced.

### Follow-up of subjects withdrawn from treatment

No follow-up is different from the normal out-of-study follow-up.

### Premature termination of the study

A decision for premature termination will take place when DSMB analysis shows that preoperative continuation of aspirin causes health dangers for the patient, for example, a significant increase of postoperative hemorrhage leading to reoperation.

## Safety reporting

### Temporary halt for reasons of subject safety

In accordance with section 10, subsection 4, of the WMO, the sponsor will suspend the study if there is sufficient ground that continuation of the study will jeopardize the subject health or safety. The sponsor will notify the accredited METC without undue delay of a temporary halt including the reason for such an action. The study will be suspended pending a further positive decision by the accredited METC. The investigator will take care that all subjects are kept informed.

### Anesthesiological safety assessment

At the request of the METC, a safety assessment of the study protocol by anesthesiologists is performed. In consultation with Dr. L. Munsterman, cardio-anesthesiologist, and the Wetenschapscommissie of the Haga Hospital, no objections were formulated to perform this study. This was supported by the argument that, except for continuing aspirin in the study group, the standard of care is not altered. Recommendation is to announce the initiation of the study in every participating center. This will be done by organizing an informative presentation to the anesthesiology team in every participating center prior to study initiation.

### AEs, SAEs, and SUSARs

#### Adverse events (AEs)

Hemorrhage-related complications such as hemorrhage itself, hemorrhage-related neurological deficits such as paresis, anesthesia and cauda syndrome, anemia, hypotension, and increased infection risk.

Thrombo-embolic complications, i.e., myocardial infarction, increase of symptomatic coronary artery disease, brain stroke, or transient ischemic attack will be reported as well.

#### Serious adverse events (SAEs)

Reoperation in case of cauda- and/or nerve compression caused by hemorrhage leading to neurological deficit. Hemorrhage leads to anemia leading to cardiovascular complications such as hypovolemic shock. These results will be reported in a study report.

#### Suspected unexpected serious adverse reactions (SUSARs)

Not applicable.

### Annual safety report

Will be provided annually to the METC and Wetenschapsbureau HMC containing all AEs, SAEs, and study progression.

### Follow-up of adverse events

All AEs will be followed until they have abated, or until a stable situation has been reached. Depending on the event, follow-up may require additional tests or medical procedures as indicated, and/or referral to the general physician or a medical specialist.

SAEs need to be reported till the end of the study within the Netherlands, as defined in the protocol.

### Data Safety Monitoring Board (DSMB)/Safety Committee

After the inclusion of 100 patients, an assessment of the Data Safety Monitoring Board will be requested for continuation of the study or suggestions for adjustment in the study protocol. In case of direct health safety issues, a direct halt of the study can be decided. The reason to appoint a DSMB is because of the invasive therapy which is needed in case of acute bleeding in the continuation group, which is the study group.

The advice(s) of the DSMB will only be sent to the sponsor of the study. Should the sponsor decide not to fully implement the advice of the DSMB, the sponsor will send the advice to the reviewing METC, including a note to substantiate why (part of) the advice of the DSMB will not be followed.

DSMB will be composed of a neurosurgeon, a hematologist, and an epidemiologist, all external and independent to the Aspin trial. The final appointment will take place after study protocol approval by the METC.

The composition of the Board will be as follows:

- Prof. Dr. M.V. Huisman; internal medicine with expertise in the field of vascular medicine at the Leids University Medical Center.
- Dr. G.J. Bouma, spinal neurosurgeon at Amsterdam University Medical Center.
- Dr. M.G.J. Gademan, clinical epidemiologist at the Leids University Medical Center.

### Adjucation commission

An adjucation commission will be assembled and assigned in order to assess the correct classification of complications. The commission will consist of a vascular internist, a cardiologist, and a neurosurgeon.

## Statistical analysis

### Co-primary study parameter(s)

Statistical analysis will be performed using SPSS. The risk of hemorrhagic complications and reduction in thrombo-embolic perioperative events within 30 days after surgery will be compared by calculating the confidence intervals of both groups and noninferiority will be claimed if the complication risk percentage stays below 6.1 percentage points.

In order to compare the effect of predefined risk factors on the primary and secondary outcome (Table 2) a multivariate regression model with an intention to treat analysis will be used.

**Table 2:** Predefined risk factors

Age	
Sex	
Type of operation Type of spinal procedure: non-instrumented decompression, fusion procedures, spinal level, degenerative, neoplastic	
Duration of surgery	
Amount of preoperative thrombocytes	
Prescribed aspirin dose (80 or 100 mg)	
Previous cardiac or cerebral infarctions (as well as coronary artery disease and transient ischemic attack)	
Duration of surgery	
Type of spinal surgery	

Additional outcomes will be the hemorrhagic outcome measures (perioperative blood loss, hemorrhage-related complications, need for reoperation, and transfusion requirement) that will be compared among both treatment groups with a chi-square or Student’s *t*-test. The treatment group bleeding risk is assumed to be below 0,0710 under the null hypothesis of inferiority.

Follow-up within the study ends at day 30 after surgery. All events after day 30 will not be registered for the Aspin trial. Incidental findings will be registered as well and assigned to the involved organ system.

In case of missing data, patients of the treating surgeon can be contacted to complete the missing information.

All efforts will be made to assemble data for the primary outcome analysis. In case this data is missing, participants will be excluded from the final analysis.

All patients randomized to one of the study arms will be analyzed according to the intention to treat analysis.

Missing data in baseline characteristics will be imputed using multiple imputation (*n*=10) based on the outcome and relevant baseline covariates using the “Multivariate Imputation by Chained Equations” (MICE) algorithm. Patients with missing primary outcomes will be excluded but every effort will be made to obtain follow-up.

The ACTS scale and PROMIS Global-10 score will be compared between pre- and postoperative assessment and between the study and control groups.

### Interim analysis

No interim-analysis will be performed. Safety examination will be performed bij DSMB at 100 inclusions. The risk of life-threatening hemorrhagic complications and irreversible cardio- and/or neuro-vascular thrombo-embolic complications will be evaluated.

## Ethical considerations

### Regulation statement

The study will be conducted according to the principles of the Declaration of Helsinki (64th World Medical Association General Assembly, Fortaleza, Brasil, October 2013) and in accordance with the Medical Research Involving Human Subjects Act (WMO).

### Recruitment and consent

This study has been approved by the medical research ethics committee; in Dutch: Medisch Ethische Toetsingscommissie (METC). Participation in this study is on a voluntary basis. If persons do not wish to participate, they can do so without specifying. Deciding not to participate in the study will not affect regular treatment and follow-up care. Participants or guardians in case are allowed to withdraw from the study at any time after they have given their written consent.

Because all persons eligible for inclusion are aged 18 and older, all eligible subjects are legally competent to decide whether they wish to participate. Persons incompetent to decide, i.e., patients with severe cognitive dysfunction or psychiatric illness are excluded from participation in the proposed study.

On the consent form, participants will be asked if they agree to use of their data should they choose to withdraw from the trial. Participants will also be asked for permission for the research team to share relevant data with people from the Universities taking part in the research or from regulatory authorities, where relevant. This trial does not involve collecting biological specimens for storage

### Benefits and risks assessment and group relatedness

Participation in this study imposes no additional risk to patients. It is unclear whether the assumed risk for an increase in hemorrhage-related complications outweighs the risk of an increase in cardiac and neurologic thrombotic perioperative events. Currently, there is no consensus on whether or not aspirin should be discontinued before cranial or spinal surgery. Aspirin is typically discontinued in cranial and spinal surgery because of a potential increased risk of hemorrhagic complications. This again might delay surgical procedures and may carry the risk of resulting in an increase in cardiac and neurologic thrombotic perioperative events.

Furthermore, a DSMB will survey the safety of the continuation of aspirin perioperatively.

There are no further disadvantages of participating in this study. Outcome measurements are recorded during admission and regular outpatient visits, and thus, do not require additional visits to the hospital.

### Compensation for injury

The sponsor/investigator has liability insurance which is in accordance with article 7, subsection 6 of the WMO.

The sponsor (also) has insurance, which is in accordance with the legal requirements in the Netherlands (Article 7 WMO and the Measure regarding Compulsory Insurance for Clinical Research in Humans of 23 June 2003). This insurance provides cover for damage to research subjects through injury or death caused by the study.

- € 750,000 (i.e., seven hundred and fifty thousand Euro) for each subject who participates in the Research;
- € 5,000,000 (i.e., five million Euro) in total for all damage incurred to all participants in all participating centers of the Research;
- € 7,500,000 (i.e., seven million and five hundred thousand Euro) for the total damage incurred by the organization for all damage disclosed by scientific research for the Sponsor as “verrichter” in the meaning of said Act in each year of insurance coverage.

The insurance applies to the damage that becomes apparent during the study or within 4 years after the end of the study.

The insurance company of this research in the Haaglanden Medical Center:

- Onderlinge Waarborgmaatschappij voor Instellingen in de Gezondheidszorg MediRisk B.A.
- Postbus 8409, 3503 RK Utrecht | Van Deventerlaan 20, 3528 AE Utrecht
- 030 - 2027280 | www.medirisk.nl
- NL14ABNA0555074757
- Inschrijving K.v.K. Midden-Nederland 30110086 | vergunning AFM 12000611

## Administrative aspects, monitoring, and publication

### Handling and storage of data and documents

Data collection and checking for quality will be performed with a study data management system (CASTOR EDC). All research data will be accessible to the clinical investigator and to the research nurse per center only and will be stored for a duration of 15 years.

After inclusion, every patient will be blinded and randomized via CASTOR EDC. Only the name of the medical center and hospital identification number will be saved to retrieve patient data.

The handling of personal data will comply with the EU General Data Protection Regulation and the Dutch Act on Implementation of the General Data Protection Regulation. (in Dutch: Uitvoeringswet AVG, UAVG).

#### Public disclosure and publication policy

Research data can be presented or published in agreement with the principal investigator and project leaders only. No research data which can be traced to individual persons will be presented or published.

### Monitoring and quality assurance

The Trial Steering group is composed of the primary investigator and the senior coordinating investigators. This group evaluates the progress of the study per 2 months.

The study monitoring plan will be composed by the counseling HMC Science Committee. The risk classification of the Aspin study is assessed by the METC as negligible. For this, the NFU guideline is handled. According to this guideline, a yearly monitor visitation per participating center will be performed.

Furthermore, an assessment of the Data and Safety Monitoring Board (DSMB) will take place after each 100 participants completes their study participation. This will happen by supplying the DSMB with an update of the data up to that time point.

### Amendments

Amendments are changes made to the research after a favorable opinion by the accredited METC has been given. All amendments will be notified to the METC that gave a favorable opinion.

All substantial amendments will be notified to the METC and to the competent authority.

Non-substantial amendments will not be notified to the accredited METC and the competent authority, but will be recorded and filed by the sponsor.

### Annual progress report

The sponsor/investigator will submit a summary of the progress of the trial to the accredited METC and the HMC Science Committee (Wetenschapsbureau) once a year. Information will be provided on the date of inclusion of the first subject, the number of subjects included and the number of subjects that have completed the trial, serious adverse events/ serious adverse reactions, other problems, and amendments.

### Temporary halt and (prematurely) end of study report

The investigator/sponsor will notify the accredited METC of the end of the study within a period of 8 weeks. The end of the study is defined as the last patient’s last visit.

The sponsor will notify the METC immediately of a temporary halt of the study, including the reason of such an action.

In case the study is ended prematurely, the sponsor will notify the accredited METC within 15 days, including the reasons for the premature termination.

Within 1 year after the end of the study, the investigator/sponsor will submit a final study report with the results of the study, including any publications/abstracts of the study, to the accredited METC.

### Public disclosure and publication policy

The results of this study will be published in an international peer-reviewed scientific journal and will be presented at (inter)national scientific conferences and meetings. This will be in accordance with the CCMO statement of publication policy.

The datasets analyzed during the current study and statistical code are available from the corresponding author on reasonable request, as is the full protocol."

### Patient public involvement

The daily organization and support of this study is provided by the Science Commission of the Haaglanden Medical Center. In all the participating centers the Science Helpdesks will be involved as well, always with the support of the Science Commission of the Haaglanden Medical Center.

## Structured risk analysis

### Potential issues of concern

- Level of knowledge about the mechanism of action

See chapter 1.

b. Previous exposure of human beings to the test product(s) and/or products with a similar biological mechanism

Aspirin is a widely used and evidence-based medicament with an antithrombotic effect. The effect is based on an irreversible inhibition of thrombocyte aggregation. This is the effect of acetylation of cyclo-oxygenase in the thrombocyte by inhibiting the prostaglandin thromboxane A2.

As described in chapter 1 no evidence is available about the, assumed, increased risk of hemorrhage-related complication peri-operatively.

c. Can the primary or secondary mechanism be induced in animals and/or in *ex-vivo* human cell material?

Not applicable.

d. Selectivity of the mechanism to target tissue in animals and/or human beings

Not applicable.

e. Analysis of potential effect

Not applicable.

f. Pharmacokinetic considerations

Not applicable

g. Study population

See chapter 4.

h. Interaction with other products

Not applicable.

i. Predictability of effect

Not applicable.

j. Can effects be managed?

In case of complications related to the perioperative continuation of aspirin, thrombocyte transfusion can be considered and, if indicated, a reoperation in order to evacuate the hematoma. In order for complications to occur related to perioperative discontinuation of aspirin diagnosis and treatment will follow according to current protocolled guidelines.

### Synthesis

Aspirin is a medication, which is widely used. In situations of emergency surgeries, experience is available where surgeries are performed without discontinuation of aspirin. In case of hemorrhage-related indication, a thrombocyte transfusion can take place, where this is not the standard of care. This gives a point of view about the need to discontinue aspirin in elective low-complex lumbar surgeries. On the other hand, one of the goals of this study is to provide evidence for the reduction or a stable presence of cardiovascular and/or neurological thromboembolic events with no increase in risk present of hemorrhage-related complications. Concerning the overall risk of life-threatening or irreversible complications in the study populations, there is no rationale to support an increase in that risk.

The remaining risks of complications in the study groups are acceptable, because the complications are known risks and manageable complications with therapies that are standard of care in current protocols. This together with the profit of the reduction of thromboembolic events as mentioned earlier.

## Data Availability

Not applicable.

## Abbreviations

ABR: General Assessment and Registration form (ABR form), the application form that is required for submission to the accredited Ethics Committee; in Dutch: Algemeen Beoordelings- en Registratieformulier (ABR-formulier)
AE: Adverse event
Aspirin: Aspirin or aspirin-like medication such as acetylsalicylic acid and carbasalate calcium
CCMO: Central Committee on Research Involving Human Subjects; in Dutch: Centrale Commissie Mensgebonden Onderzoek
CV: Curriculum Vitae
CVD: Cardiovascular diseases
DSMB: Data Safety Monitoring Board
EU: European Union
IC: Informed Consent
LUmc: Leiden University Medical Center
METC: Medical research ethics committee (MREC); in Dutch: medisch-ethische toetsingscommissie (METC)
(S)AE: (Serious) adverse event
Sponsor: The sponsor is the party that commissions the organization or performance of the research, for example, a pharmaceutical company, academic hospital, scientific organization, or investigator. A party that provides funding for a study but does not commission it is not regarded as the sponsor, but referred to as a subsidizing party
UAVG: Dutch Act on Implementation of the General Data Protection Regulation; in Dutch: Uitvoeringswet AVG
WMO: Medical Research Involving Human Subjects Act; in Dutch: Wet Medisch-wetenschappelijk Onderzoek met Mensen
